# Supplementary figures and images for: One and the same? How similar are basic human values and economic preferences
Source: PLoS One. 2024 Feb 15;19(2):e0296852. doi: 10.1371/journal.pone.0296852 (PMC10868778; doi:10.1371/journal.pone.0296852)

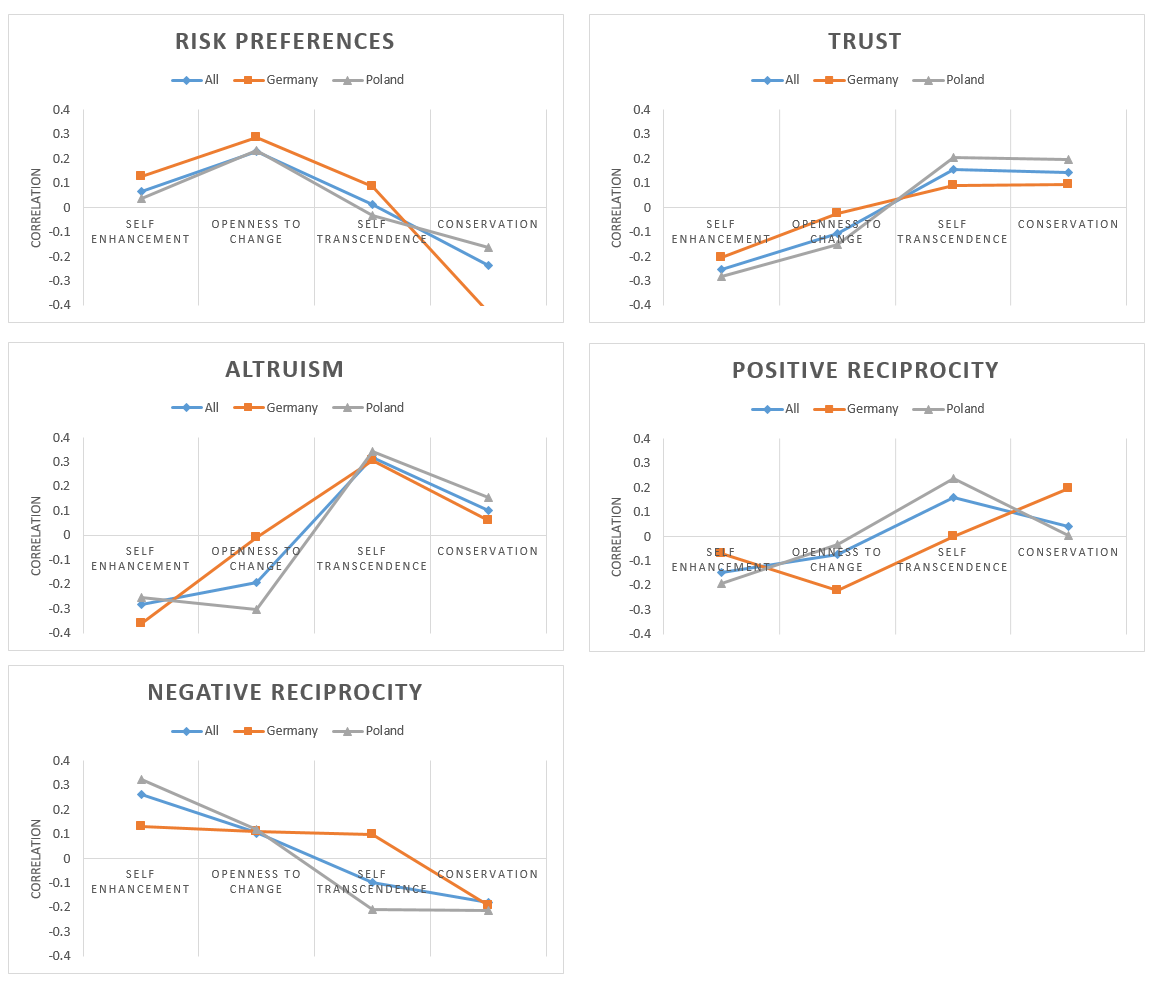

Supplement: S1 Fig — (PNG) [file pone.0296852.s001.png]

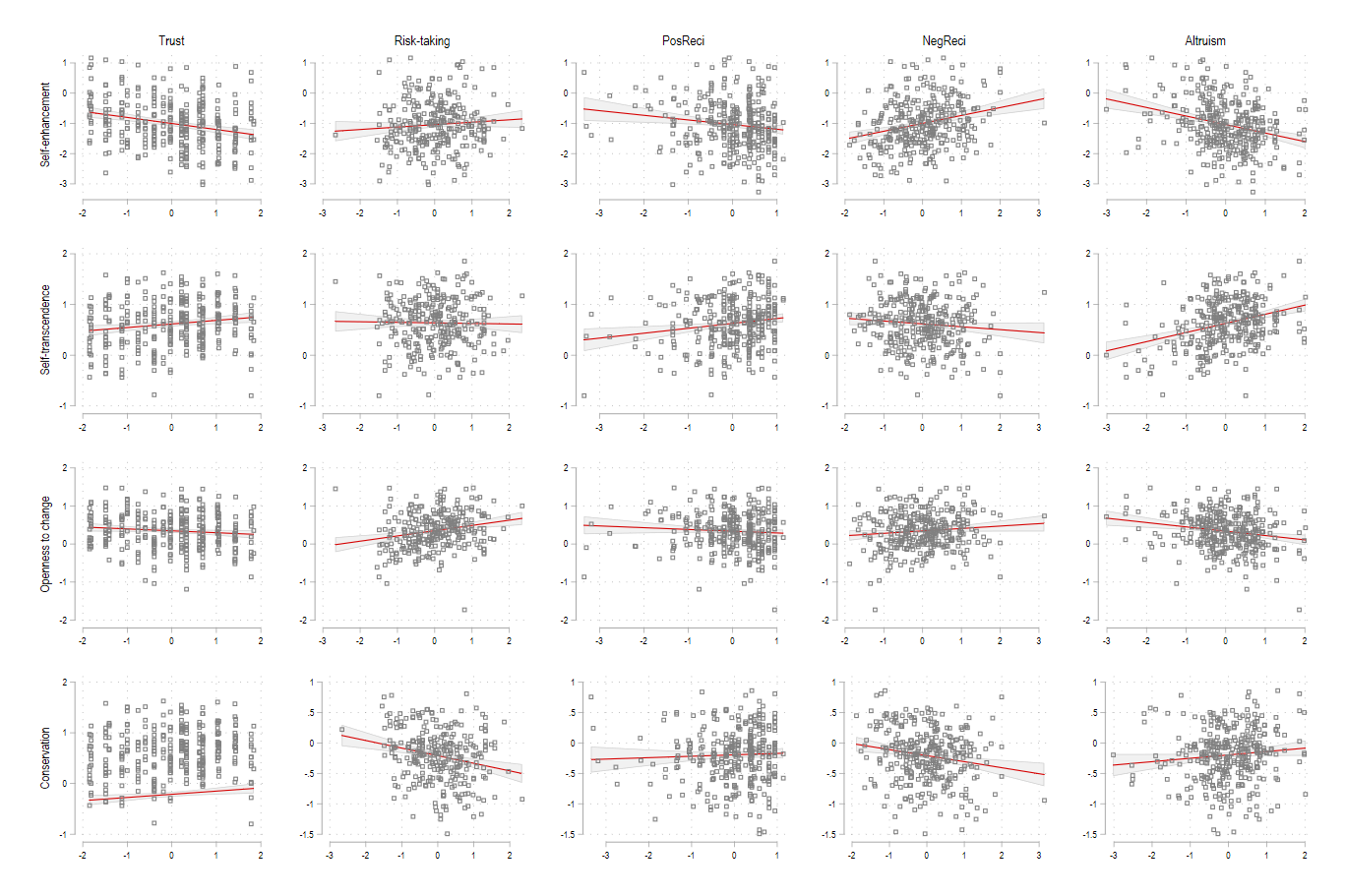

Supplement: S2 Fig — Scatter plot on values and economic preference. Grey squares represent observations, red line linear fit regression lines, grey shaded area 95% confidence interval. (PNG) [file pone.0296852.s002.png]
