# Supplementary material for: One and the same? How similar are basic human values and economic preferences
Source: PLoS One. 2024 Feb 15;19(2):e0296852. doi: 10.1371/journal.pone.0296852 (PMC10868778; doi:10.1371/journal.pone.0296852)
Supplement: S1 Table — (PDF) [file pone.0296852.s003.pdf]

S1 Table. Preregistered hypotheses on first-order values.

Higher-order value	Lower-level value	Risk taking	Trust	Altruism	Positive rec.	Negative rec.
**Self-enhancement**	Power		-	-	+	+
	Achievement			-	+	
**Openness to change**	Stimulation	+				
	Hedonism					
	Self-Direction	+				
**Self-transcendence**	Universalism		+	+	+	(-)
	Benevolence			+	+	
**Conservation**	Conformity	-			+	+
	Tradition	-			+	+
	Security	-			+	+
