# Supplementary material for: One and the same? How similar are basic human values and economic preferences
Source: PLoS One. 2024 Feb 15;19(2):e0296852. doi: 10.1371/journal.pone.0296852 (PMC10868778; doi:10.1371/journal.pone.0296852)
Supplement: S2 Table — (PDF) [file pone.0296852.s004.pdf]

S2 Table. Descriptive Statistics of the Specific Lower-Level Values.

Variable	*N*	*Mean*	*Std.*	*Min*	*Max*
Achievement	336	0.02	0.82	-2.81	1.77
Hedonism	337	0.29	0.78	-2.47	2.00
Stimulation	338	-0.35	0.93	-2.84	1.91
Self-Direction Action	337	0.78	0.63	-1.81	2.89
Self-Direction Thought	337	0.62	0.69	-1.47	3.13
Universalism Tolerance	337	0.67	0.73	-1.84	2.41
Universalism Nature	338	0.14	0.93	-3.04	2.04
Universalism Concern	337	0.63	0.77	-2.14	2.11
Benevolence Care	336	0.82	0.57	-0.96	2.67
Benevolence Dependability	337	0.87	0.58	-1.00	2.23
Humility	337	-0.41	0.93	-3.44	2.13
Conformity Interpersonal	337	-0.21	0.97	-3.49	2.13
Conformity Rules	337	-0.42	0.99	-3.33	3.47
Tradition	338	-0.78	1.33	-3.49	2.19
Security Society	338	0.36	0.75	-2.44	2.56
Security Personal	337	0.06	0.70	-2.95	1.95
Face	337	0.03	0.88	-2.69	2.16
Power Resources	337	-1.44	1.13	-4.11	1.36
Power Dominance	338	-1.68	1.14	-4.29	2.81
