# Supplementary material for: One and the same? How similar are basic human values and economic preferences
Source: PLoS One. 2024 Feb 15;19(2):e0296852. doi: 10.1371/journal.pone.0296852 (PMC10868778; doi:10.1371/journal.pone.0296852)
Supplement: S3 Table — (PDF) [file pone.0296852.s005.pdf]

S3 Table. Descriptive statistics – Polish sample.

Variable	*N*	*Mean*	*Std.*	*Min*	*Max*
Sociodemographics					
Age	211	34.08	12.75	17.00	84.00
Gender (female = 1, male = 2)	211	1.42	0.50	1.00	2.00
Income	207	1.76	0.60	1.00	4.00
Higher-order values (centered around the individual mean)					
Self-Enhancement	211	-1.06	0.88	-3.26	1.11
Self-Transcendence	211	0.52	0.46	-0.79	1.86
Openness to Change	211	0.23	0.48	-1.72	1.48
Conservation	211	-0.05	0.39	-1.49	0.84
Economic preferences (standardized based on the instructions in Falk et al. [8])					
Risk taking	208	0.00	0.78	-1.67	1.91
Positive Reciprocity	209	0.00	0.76	-3.35	0.94
Negative Reciprocity	201	0.00	0.81	-1.89	1.99
Altruism	211	0.00	0.83	-2.51	1.98
Trust	200	0.00	1.00	-1.85	1.78
