# Supplementary material for: One and the same? How similar are basic human values and economic preferences
Source: PLoS One. 2024 Feb 15;19(2):e0296852. doi: 10.1371/journal.pone.0296852 (PMC10868778; doi:10.1371/journal.pone.0296852)
Supplement: S4 Table — (PDF) [file pone.0296852.s006.pdf]

S4 Table. Descriptive statistics – German sample.

Variable	*N*	*Mean*	*Std.*	*Min*	*Max*
Sociodemographics					
Age	120	21.32	3.57	17.00	38.00
Gender (female = 1, male = 2)	120	1.31	0.48	1.00	3.00
Income	113	1.78	0.74	1.00	4.00
Higher-order values (centered around the individual mean)					
Self-Enhancement	119	-1.02	0.69	-2.38	1.18
Self-Transcendence	119	0.83	0.41	-0.22	1.63
Openness to Change	119	0.52	0.46	-0.95	1.46
Conservation	119	-0.45	0.43	-1.46	0.86
Economic preferences (*standardized based on the instructions in Falk et al. [8])*					
Risk Taking	120	-0.01	0.80	-2.73	2.41
Positive Reciprocity	119	0.06	0.68	-2.75	1.14
Negative Reciprocity	118	-0.03	0.77	-1.67	3.14
Altruism	120	0.00	0.81	-3.02	2.01
Trust	115	0.01	1.01	-1.82	1.84
