# Supplementary material for: One and the same? How similar are basic human values and economic preferences
Source: PLoS One. 2024 Feb 15;19(2):e0296852. doi: 10.1371/journal.pone.0296852 (PMC10868778; doi:10.1371/journal.pone.0296852)
Supplement: S5 Table — Pearson correlation coefficients (top) and significance levels (bottom). (PDF) [file pone.0296852.s007.pdf]

S5 Table. Variable Correlations

		1	2	3	4	5	6	7	8	9
1	Trust	1.000								
2	Risk Preferences	0.098	1.000							
		0.080								
3	Positive Reciprocity	0.030	0.106	1.000						
		0.597	0.054							
4	Negative Reciprocity	-0.067	0.102	-0.019	1.000					
		0.235	0.066	0.733						
5	Altruism	0.277	0.098	0.157	0.068	1.000				
		0.000	0.072	0.004	0.218					
6	Self-Enhancement	-0.251	0.065	-0.145	0.263	-0.284	1.000			
		0.000	0.235	0.008	0.000	0.000				
7	Self-Transcendence	0.155	0.012	0.160	-0.097	0.318	-0.616	1.000		
		0.006	0.825	0.003	0.081	0.000	0.000			
8	Openness to Change	-0.106	0.232	-0.073	0.105	-0.192	0.095	0.089	1.000	
		0.059	0.000	0.182	0.057	0.000	0.082	0.103		
9	Conservation	0.144	-0.237	0.041	-0.181	0.103	-0.369	-0.311	-0.743	1.000
		0.010	0.000	0.460	0.001	0.060	0.000	0.000	0.000	
