# Supplementary material for: One and the same? How similar are basic human values and economic preferences
Source: PLoS One. 2024 Feb 15;19(2):e0296852. doi: 10.1371/journal.pone.0296852 (PMC10868778; doi:10.1371/journal.pone.0296852)
Supplement: S6 Table — The dependent variables are in the columns. Heteroscedasticity robust standard errors are in brackets. Significance levels: * p < 0.1, ** p < 0.05, *** p < 0.01. Females were the reference category for gender, the variable “Other Nationality” represents individuals who were not country nationals, with Polish nationals as the reference category. PosReci = positive reciprocity; NegReci = negative reciprocity; N = sample size. (PDF) [file pone.0296852.s008.pdf]

S6 Table. OLS regression coefficients with all socio-demographics as control variables

	Risk Taking		Trust		Altruism		PosReci		NegReci	
Openness to Change		0.17								
		(0.18)								
Conservation		-0.41**						0.01		-0.14
		(0.20)						(0.13)		(0.18)
Self Transcendence				-0.00		0.51***				0.12
				(0.18)		(0.15)				(0.19)
Self Enhancement				-0.24**		-0.04		-0.11		0.29***
				(0.10)		(0.08)		(0.07)		(0.11)
Age	0.00	0.01*	0.02***	0.01**	0.01***	0.01	0.00	0.00	-0.00	0.00
	(0.00)	(0.00)	(0.01)	(0.01)	(0.00)	(0.00)	(0.00)	(0.00)	(0.00)	(0.00)
Male	0.23**	0.20**	-0.15	-0.07	-0.33***	-0.18*	-0.09	-0.05	0.14	0.05
	(0.09)	(0.09)	(0.12)	(0.12)	(0.10)	(0.10)	(0.09)	(0.09)	(0.09)	(0.10)
Other Gender	-0.71***	-0.78***	-0.88***	-0.60***	-2.53***	-2.32***	-2.16***	-2.04***	-1.51***	-1.82***
	(0.13)	(0.13)	(0.14)	(0.17)	(0.10)	(0.13)	(0.10)	(0.12)	(0.14)	(0.15)
German	0.08	-0.09	0.25*	0.18	0.12	-0.11	0.11	0.08	-0.05	-0.07
	(0.11)	(0.12)	(0.14)	(0.15)	(0.11)	(0.12)	(0.10)	(0.11)	(0.10)	(0.11)
Other Nationality	0.21	0.14	-0.07	-0.12	-0.07	-0.10	-0.84*	-0.83	0.28	0.22
	(0.34)	(0.35)	(0.39)	(0.40)	(0.23)	(0.19)	(0.50)	(0.51)	(0.36)	(0.34)
Income	0.06	0.01	-0.10	-0.11	-0.05	-0.08	0.09	0.09	0.12	0.10
	(0.07)	(0.07)	(0.08)	(0.08)	(0.06)	(0.06)	(0.06)	(0.06)	(0.07)	(0.07)
Constant	-0.28	-0.41**	-0.46*	-0.50**	-0.20	-0.29	-0.25	-0.28	-0.13	-0.07
	(0.20)	(0.19)	(0.25)	(0.24)	(0.21)	(0.21)	(0.21)	(0.21)	(0.19)	(0.19)
N	324	324	308	308	326	326	322	322	315	315
R	0.02	0.10	0.06	0.09	0.10	0.17	0.06	0.07	0.03	0.10
