# Supplementary material for: One and the same? How similar are basic human values and economic preferences
Source: PLoS One. 2024 Feb 15;19(2):e0296852. doi: 10.1371/journal.pone.0296852 (PMC10868778; doi:10.1371/journal.pone.0296852)
Supplement: S7 Table — OLS regression results using top row variables as dependent variables. Heteroscedasticity robust standard errors are in brackets. Significance levels: * p < 0.1, ** p < 0.05, *** p < 0.01. Variable names: UNC–Universalism concern, UNN–Universalism nature, UNT–Universalism tolerance, POD–Power dominance, POR–Power resources, BEC–Benevolence care, BED–Benevolence dependability, AC–Achievement, SEP—Security personal, SES–Security societal, COR–Conformity rules, COI–Conformity interpersonal, TR–Tradition, ST–Stimulation, SDA–Self-direction action, SDT–Self-direction thought. (PDF) [file pone.0296852.s009.pdf]

S7 Table. Predicting Behavioral Preferences - Lower Level Values

	Trust		Altruism		Risk Taking		Posreci		NegReci	
UNC	0.027	0.042	0.114	0.135			0.159**	0.162**	0.172**	0.186**
	(0.097)	(0.102)	(0.078)	(0.086)			(0.068)	(0.068)	(0.068)	(0.072)
UNN	0.029	0.036	0.092*	0.121**			-0.071	-0.099*	0.016	0.012
	(0.073)	(0.073)	(0.054)	(0.055)			(0.049)	(0.052)	(0.056)	(0.060)
UNT	0.155*	0.184*	-0.016	0.002			0.031	0.032	-0.241***	-0.230***
	(0.093)	(0.099)	(0.068)	(0.071)			(0.071)	(0.075)	(0.067)	(0.071)
POD	-0.084	-0.024	-0.050	0.017					0.099*	0.123**
	(0.062)	(0.067)	(0.046)	(0.048)					(0.051)	(0.053)
POR	-0.092	-0.092	-0.071	-0.112**					0.072	0.041
	(0.062)	(0.067)	(0.052)	(0.054)					(0.047)	(0.050)
BEC			0.236**	0.258***			0.094	0.097		
			(0.095)	(0.095)			(0.090)	(0.091)		
BED			-0.050	-0.045			0.082	0.105		
			(0.089)	(0.088)			(0.093)	(0.094)		
AC			-0.019	0.070						
			(0.058)	(0.064)						
SEP					-0.179**	-0.181**	-0.082	-0.088	-0.047	-0.030
					(0.072)	(0.076)	(0.061)	(0.061)	(0.073)	(0.075)
SES					-0.031	-0.042	0.068	0.068	0.063	0.049
					(0.067)	(0.068)	(0.064)	(0.064)	(0.065)	(0.066)
COR					0.028	0.017	-0.016	-0.010	0.033	0.051
					(0.048)	(0.052)	(0.060)	(0.063)	(0.048)	(0.051)
COI					-0.011	-0.007	0.123**	0.122**	-0.182***	-0.169***
					(0.046)	(0.048)	(0.049)	(0.052)	(0.048)	(0.050)
TR					0.095***	0.084**	0.033	0.004	-0.111***	-0.144***
					(0.033)	(0.039)	(0.036)	(0.045)	(0.040)	(0.047)
ST					0.213***	0.211***				
					(0.050)	(0.055)				
SDA					0.191**	0.192**				
					(0.094)	(0.098)				
SDT					-0.014	-0.028				
					(0.077)	(0.076)				
Controls	No	Yes	No	Yes	No	Yes	No	Yes	No	Yes
N	319	307	334	322	331	320	330	318	323	312
R	0.07	0.10	0.13	0.18	0.15	0.16	0.08	0.12	0.18	0.18

* $p < 0.1$, ** $p < 0.05$, *** $p < 0.01$. Standard errors in parentheses.
