# Supplementary material for: One and the same? How similar are basic human values and economic preferences
Source: PLoS One. 2024 Feb 15;19(2):e0296852. doi: 10.1371/journal.pone.0296852 (PMC10868778; doi:10.1371/journal.pone.0296852)
Supplement: S8 Table — (PDF) [file pone.0296852.s010.pdf]

S8 Table. Measurement invariance of higher-order values across Germany and Poland

	χ^2	df	CFI	RMSEA	SRMR
**Self-enhancement**					
Configural	104.85	48	.950	.084 [.062-.106]	.057
Metric	121.02	54	.942	.086 [.065-.106]	.072
Scalar	241.30	60	.842	.134 [.116-.152]	.128
Partial scalar (PVQ6 PVQ32)	122.65	58	.944	.081 [.061-.101]	.072
**Self-transcendence**					
Configural	272.82	160	.932	.065 [.051-.078]	.055
Metric	293.52	170	.925	.066 [.053-.078]	.106
Scalar	374.68	180	.882	.080 [.069-.091]	.117
Partial scalar (released PVQ8 PVQ11 PVQ14)	318.15	177	.915	.069 [056-.081]	.105
**Conservation**					
Configural	529.54	336	.913	.058 [.049-.068]	.064
Metric	536.98	350	.919	.056 [.047-.068]	.069
Scalar	635.29	364	.883	.066 [.058-.075]	.078
Partial scalar (released PVQ22, PVQ40, PVQ49)	582.00	361	.904	.060 [.051-.069]	.072
**Openness to change**					
Configural	192.73	96	.911	.077 [.061-.093]	.065
Metric	263.67	106	.855	.094 [.080-.108]	.120
Partial metric (released in HE-ST: PVQ46, PVQ10, PVQ43; released in SAD: PVQ36)	207.37	102	.903	.078 [.063-.093]	.082
Scalar	347.60	111	.783	.112 [.099-.126]	.137
Partial scalar (released PVQ10 PVQ28 PVQ30 PVQ43 PVQ56)	218.88	106	.896	.079 [.064-.094]	.087
