# Supplementary material for: One and the same? How similar are basic human values and economic preferences
Source: PLoS One. 2024 Feb 15;19(2):e0296852. doi: 10.1371/journal.pone.0296852 (PMC10868778; doi:10.1371/journal.pone.0296852)
Supplement: S1 Text — (PDF) [file pone.0296852.s011.pdf]

S1 Text. Country Differences.

In a final analysis for robustness, we looked more closely into country differences in the associations between economic preferences and human values. For risk preferences, the correlations were similar across countries. For trust, the correlations had the same sign, but they were somewhat lower for German participants. For altruism, the relations closely matched across countries with the exception of openness to change, which was uncorrelated in Germany but negatively correlated in Poland. For positive and negative reciprocity, the signs of the associations were mostly either the same or negligible. The only deviations were found for self-transcendence and negative reciprocity. In Germany, self-transcendence was not significantly correlated to negative reciprocity ($r = 0.10$, $p = 0.28$) while it was negatively correlated in Poland ($r = -0.21$, $p=0.03$).
