# Supplementary material for: One and the same? How similar are basic human values and economic preferences
Source: PLoS One. 2024 Feb 15;19(2):e0296852. doi: 10.1371/journal.pone.0296852 (PMC10868778; doi:10.1371/journal.pone.0296852)
Supplement: Attachment — Submitted filename: Review report for PlosOne.pdf [file pone.0296852.s012.pdf]

Review report for PONE-D-23-03410, "One and the same? How similar are basic human values and behavioral economic preferences"

This manuscript attempts to address an interesting research question. Because psychologists measure basic human values and economists measure preferences, the authors would like to see whether values and preferences are similar and study the possible mechanism underlying the similarity.

To do that, they use surveys to measure mainly four values (openness to change, conservation, self-transcendence, and self-enhancement) and five preferences (trust, risk preferences, positive reciprocity, negative reciprocity, and altruism) of 339 participants in Poland and Germany and proceed in three steps.

In the first step, the authors calculate the correlation coefficients between each individual value and each preference. Not surprisingly some correlations are significant (for instance, openness to change is positively correlated with risk taking) but others are not. In the second step, the authors regress preferences on values but at the same time, they control for some sociodemographic characteristics. Again, not surprisingly, some values can explain preferences even when the sociodemographic characteristics are controlled for, but others cannot. Lastly, they offer a visualization regarding how each particular preference is close to a particular value. Overall, the research question is interesting, but the results are not surprising. Presumably, even if we just focus on preferences, we may very likely arrive at the same conclusion as some preferences may be more similar to each other (for instance, trust and altruism are quite similar) and others are less so. Hence, at the end of the day, it is not clear what additional insight we have learned from this research. The mechanism, which interests me the most when reading the abstract, is lacking.

With that said, because Plos One only asks for scientifically valid research, as far as this is concerned, with some revisions, the manuscript would meet the publication requirement.

I now bullet list comments/questions/suggestions I have. The manuscript does not number pages, but page number should be easily inferred.

(1) Throughout the manuscript, the authors more often than not use the term behavioral economics in a rather unusual way. For instance, risk preferences are quite classical preferences, it is unusual to describe them as behavioral. The term "behavioral"

often refers to preferences that are not classical and hence insights from psychology or cognitive neuroscience are used to motivate why economists should care about them.

(2) On page 3, second paragraph, line 5, the sentence "While the values approach is characterized by a top-down approach, thinking about the highest possible motivation level people can have that then influences behavior, the behavioral economic approach operates in the opposite way" sounds strange. Please check your grammar. The next sentence, "It starts from the behavior and tries to infer the preference structure using a revealed choice approach" has a rather unusual way of describing what economists do. Economists infer preference from choice, this inference is called the revealed preference approach (not revealed choice approach). Choice is the data of economists; it is preference that gets revealed from choice. For economists, preference is the underlying driver of choice much like values drive behavior for psychologists. Since the authors use the Global Preference Survey (GPS) developed by Falk et al. and there are survey questions that directly ask about preferences, it is not clear there is indeed such a difference between these two approaches.

(3) On page 6, why is the section titled "Behavioral economic preferences" shorthanded as BPP? Again, the use of the term "behavioral" is unusual. Even Falk did not call the survey module behavioral.

(4) On page 6, 9th line from the bottom, "the risk premium" probably should be "the risk premium of stocks."

(5) On page 8, 7th line from the bottom, the word "I" should be removed.

(6) On page 12, now BPP refers to "behavioral economic preferences parameters." In this paragraph, the authors claim the complete survey and item construction can be found in the Supporting Information (in English). It is not included there. Also, the authors claim they will make data and the syntax available on osf.

(7) On page 13, the authors pooled data from Poland and Germany together. A formal statistical test of no difference is needed before data from the two countries can be pooled.

(8) The authors should keep the reported values to the same decimal places. Sometimes they report up to 2, other times to 3.

(9) The authors should make sure the values they reported in the table are the same as what they reported in the text. Just to give one example, on page 15, 2nd line, they reported an r=-0.283 but the corresponding number in Table 3 is -0.284. There are too many inconsistencies. The burden should fall on the authors, not on the readers.

(10) When the authors said there is a weak correlation, do they base the claim on a small r or a small p-value? The authors should explain their criterion and stick to it

consistently throughout.

(11) The authors did not report the results for H7 and H8. But in fact, positive reciprocity does correlate with self-transcendence. Why do they ignore this result? Positive reciprocity also negatively correlates with self-enhancement, by the way.

(12) The author should justify why they do not do multiple comparison corrections for the results they report in Table 3. If the corrections are made, some significant results may become insignificant.

(13) Shaded color should be explained in the legends of Table 3.

(14) GPS does include preferences on patience. The authors do not mention patience throughout the manuscript but somehow there is a column on patience in Table 4.

(15) On page 20, the last paragraph the authors seem to expect that results in step 2 would be similar to those in step 1. I do not understand their logic. They only calculate correlation coefficients in step 1. But in step 2, they run multiple variable regressions. Logically there is no guarantee that one implies the other. So why should one expect to see similar results in both steps? Because the results are not robust, the authors should address the conceptual difference among the three steps to help the readers understand which step best addresses their research curiosity.

(16) Table A5 is confusing. I don't understand what "Same Sample all" means though I did notice that the sample size is fixed at 296.

(17) The title of Table A6 should be changed. The authors are regressing values on preferences here.

(18) The shorthands for lower-level values in Table A8 should be explained.

(19) On page 39, several p-values and table number are missing (see XXX and x).

(20) Currently all figures are called Fig. 1. See pages 43, 44.

(21) Again, the shorthands for lower-level values on page 44 should be explained.

Overall, the manuscript is not written with enough care. The authors should read the manuscript before they submit their revised version.
