# Supplementary material for: One and the same? How similar are basic human values and economic preferences
Source: PLoS One. 2024 Feb 15;19(2):e0296852. doi: 10.1371/journal.pone.0296852 (PMC10868778; doi:10.1371/journal.pone.0296852)
Supplement: Attachment — Submitted filename: Reviewers comments and how we addressed them_final.docx [file pone.0296852.s013.docx]

Reviewers’ comments and how we addressed them

Editor

| Comment | How we addressed it |
| --- | --- |
| 1. Two reviewers had issues with the motivation of the paper. Please strengthen this part by providing some rationale for why it is important to compare the values approach (top-down) with the behavioral economics approach (bottom-up) . | Researchers sometimes work in peaceful co-existence instead of building on previous theories (Karl Dieter Opp, personal communication). They may use similar concepts which have different names. Thus, it is fundamental to determine whether economic preferences and basic human values, two very widely used theories, include similar concepts with different names and measures. If not, they may prove to be complementary for explaining behavior. We have now added some discussion in the paper on why it is important to examine relations between concepts in the two theories.  The following has been added to the abstract:  “Could it be that they represent similar motivations, that is, that they are one and the same and mutually exclusive, or are they rather complementary? Finding that they are rather complementary may suggest that combining them to explain behavior might be beneficial.”  We added several strands of arguments to the motivation and discussion of the paper. The first is related to identifying the underlying driver of an attitude or a behavior which requires a better understanding of the relation between values and preferences. The second is related to the debate around external validity of findings in the economic literature. |
| 2. Especially, at the conceptual level, one the reviewers is especially critical of how the paper uses the term “behaviroal“ in a very unconventional sense. Please also offer some justifications or make revisions accordingly. | The term for preferences come from the original economic literature, but they were complemented in the Global Preference Survey by Falk et al. by measures that are less often used in economic research (like positive and negative reciprocity). Therefore, we had originally called them behavioral economic preferences. But considering the comments by the reviewers, we have now renamed them “economic preferences” throughout the paper (vs. psychological basic human values). |
| 3. All reviewers raised concerns about the sample characteristics of your studies. For example, since they are not incentivized economic experiments, why did you simply recruit students? | We have now added an explanation to address this point. Since we are not interested in descriptives but rather in associations, it may be justifiable to use convenience samples, for example with students in Germany. To account for potential bias, we examined whether the results hold in the two different samples in Germany and Poland. Furthermore, in our regression analyses we controlled for sociodemographic characteristics (including nationality) to account for potential compositional effects. Results in the two countries, and with or without sociodemographic variables, were essentially similar. |
| 4. Since the data was collected from Germany and Poland, is there a systematic difference between these two country samples? Can they be simply pooled for analysis? | We used two countries rather than just one to have better external validity for our findings. Indeed, we reported results for the pooled data, because the findings did not differ fundamentally across the samples, and to simplify the report. However, we agree that a formal test makes sense. We performed Manova tests for group differences on the main variables of concern, see footnote 9. Whereas values differed across samples, economic preferences did not. However, we also did a test for measurement invariance for values and can show that we have full metric and partial scalar invariance (see Annex table A8 for full table). Also, conclusions when running the models separately were essentially similar (available in the open data link, see below). We also controlled for nationality in the regression models (Table 4) to make sure we do not make our results biased due to this pooling. |
| 5. Analytically, all reviewers raised several questions about both the statistical strategies and the paper’s explosion of the results. Please provide more detailed justifications for your methods and improve how you report the results to avoid potential confusions. | Mainly in the body of the text but also in the Appendix, we have now reduced the number of tables with results to the minimum to reduce confusion. We have now also tried to extend the explanation of the choice of methods. |
| 6. There are also quite a few minor suggestions made by all the reviewers. Please also address them properly. | Done, see specific answers below. |
| 7. Please ensure that your manuscript meets PLOS ONE's style requirements, including those for file naming. | Yes |
| 8. Please ensure that you include a title page within your main document. Could you therefore please include the title page into the beginning of your manuscript file itself, listing all authors and affiliations. | Done. |
| 9. We note that Figure 1 in your submission contain copyrighted images. All PLOS content is published under the Creative Commons Attribution License (CC BY 4.0), which means that the manuscript, images, and Supporting Information files will be freely available online, and any third party is permitted to access, download, copy, distribute, and use these materials in any way, even commercially, with proper attribution. We require you to either (1) present written permission from the copyright holder to publish these figures specifically under the CC BY 4.0 license, or (2) remove the figures from your submission. Alternatively, supply a replacement figure that complies with the CC BY 4.0 license. Please check copyright information on all replacement figures and update the figure caption with source information. If applicable, please specify in the figure caption text when a figure is similar but not identical to the original image and is therefore for illustrative purposes only. | Indeed, we do not own the rights to the figure. After some consideration we deleted the figure from the paper as the paper does not require a visual representation of the theory. |
| 10. In your Data Availability statement, you have not specified where the minimal data set underlying the results described in your manuscript can be found. PLOS defines a study's minimal data set as the underlying data used to reach the conclusions drawn in the manuscript and any additional data required to replicate the reported study findings in their entirety. All PLOS journals require that the minimal data set be made fully available. For more information about our data policy, please see [http://journals.plos.org/plosone/s/data-availability](https://urldefense.com/v3/__http:/journals.plos.org/plosone/s/data-availability__;!!DOxrgLBm!Htn9OZd5DU9hnhvWhPRWswfmf3x7fNhV4izk2yChYyRcdte212zSMOTkFPrF-51zUuKcAPGQ2fTa52diqwoxDx9GDWZGc568o33ZeQ$). Upon re-submitting your revised manuscript, please upload your study’s minimal underlying data set as either Supporting Information files or to a stable, public repository and include the relevant URLs, DOIs, or accession numbers within your revised cover letter. For a list of acceptable repositories, please see [http://journals.plos.org/plosone/s/data-availability#loc-recommended-repositories](https://urldefense.com/v3/__http:/journals.plos.org/plosone/s/data-availability*loc-recommended-repositories__;Iw!!DOxrgLBm!Htn9OZd5DU9hnhvWhPRWswfmf3x7fNhV4izk2yChYyRcdte212zSMOTkFPrF-51zUuKcAPGQ2fTa52diqwoxDx9GDWZGc56ars4Pfg$). Any potentially identifying patient information must be fully anonymized. | We have made the data set for all analysis including the codes of the analyses and the questionnaires available anonymously here (also mentioned in the paper): <https://osf.io/v3kqt/?view_only=69213a56704647ae910a10cc70fcada5>  (Upon publication, the data can be de-anonymized) |
| 11. We note that you have stated that you will provide repository information for your data at acceptance. Should your manuscript be accepted for publication, we will hold it until you provide the relevant accession numbers or DOIs necessary to access your data. If you wish to make changes to your Data Availability statement, please describe these changes in your cover letter and we will update your Data Availability statement to reflect the information you provide. | See answer above. |
| 12. Please include a separate caption for each figure in your manuscript. | We have done so and also added page numbers to the manuscript for easier reference.  To Do: in final submission, need to make sure this is fixed as we have captions in the text and captions in the separate figure files. |

Reviewer 1

| Comment | How we addressed it |
| --- | --- |
| 1. Overall, the research question is interesting, but the results are not surprising. Presumably, even if we just focus on preferences, we may very likely arrive at the same conclusion as some preferences may be more similar to each other (for instance, trust and altruism are quite similar) and others are less so. Hence, at the end of the day, it is not clear what additional insight we have learned from this research. The mechanism, which interests me the most when reading the abstract, is lacking. | We have now added a discussion of the added value: What do we learn from the study? Are values and economic preferences mutually exclusive or rather complementary? If rather complementary, it may be useful to combine them for a better theory-driven behavioral explanation. We discussed this issue, added mechanisms in more detail (also in the abstract) and expanded on the implications of the findings in the last section. |
| 2. Throughout the manuscript, the authors more often than not use the term behavioral economics in a rather unusual way. For instance, risk preferences are quite classical preferences, it is unusual to describe them as behavioral. The term “behavioral” often refers to preferences that are not classical and hence insights from psychology or cognitive neuroscience are used to motivate why economists should care about them. | This is a correct and a valuable comment. The term for preferences comes from the economic literature, but the measures we use were complemented in the Global Preference Survey by Falk et al. by measures that are rarely used in standard economic research (like positive and negative reciprocity). Therefore, we had originally called them behavioral economic preferences to try to make this clear. However, this may have led to confusion. We have now therefore renamed them “economic preferences”. We have also added a footnote explaining that they are not merely economic preferences, but for the purposes of this paper we will stick with this terminology. |
| 3. On page 3, second paragraph, line 5, the sentence “While the values approach is characterized by a top-down approach, thinking about the highest possible motivation level people can have that then influences behavior, the behavioral economic approach operates in the opposite way” sounds strange. Please check your grammar. | We have reworded the sentence to make it easier to understand.  Values are derived in a top-down approach, thinking about the highest possible motivation level people can have, which can then translate into behavior. In contrast, the economic approach operates in the opposite way, by revealing the preferences from behavior. |
| 4. The next sentence, “It starts from the behavior and tries to infer the preference structure using a revealed choice approach” has a rather unusual way of describing what economists do. Economists infer preference from choice, this inference is called the revealed preference approach (not revealed choice approach). Choice is the data of economists; it is preference that gets revealed from choice. For economists, preference is the underlying driver of choice much like values drive behavior for psychologists. Since the authors use the Global Preference Survey (GPS) developed by Falk et al. and there are survey questions that directly ask about preferences, it is not clear there is indeed such a difference between these two approaches. | We have now tried to shorten the explanation on the revealed preference approach: “In contrast, the economic approach operates in the opposite way, starting from the behavior and trying to infer the preference structure using a preference approach revealed through the choices people make.”  However, regarding the criticism of the survey method, this is only partially true. In contrast to lab experiments where only choices are measured, the survey asks people directly both about their direct preferences and their choices (e.g. using a staircase method on which lottery choices people would accept or for reciprocity on how much money to give to a stranger, from which risk preferences are inferred). |
| 5. On page 6, why is the section titled “Behavioral economic preferences” shorthanded as BPP? Again, the use of the term “behavioral” is unusual. Even Falk did not call the survey module behavioral. | See response above: changed to “economic preferences” throughout the whole manuscript. |
| 6. On page 6, 9th line from the bottom, “the risk premium” probably should be “the risk premium of stocks.” | This is correct, we have changed the text accordingly. |
| 7. On page 8, 7th line from the bottom, the word “I” should be removed. | Corrected, thanks for pointing out the mistake. |
| 8. On page 12, now BPP refers to “behavioral economic preferences parameters.” | See comment above: changed to “economic preferences” throughout the whole manuscript. |
| 9. In this paragraph, the authors claim the complete survey and item construction can be found in the Supporting Information (in English). It is not included there. Also, the authors claim they will make data and the syntax available on osf. | The questionnaires and the data for the study can be found here: <https://osf.io/v3kqt/?view_only=69213a56704647ae910a10cc70fcada5>  We include the German and Polish original questionnaire and the automatically translated English one (deepl). |
| 10. On page 13, the authors pooled data from Poland and Germany together. A formal statistical test of no difference is needed before data from the two countries can be pooled. | Done. Indeed, we pooled the data to simplify the report. However, we agree that a formal test of no difference is necessary. We performed invariance tests and Manova tests for group differences on the main variables of concern, now reported in the results section, footnote 9. We find full metric and partial scalar variance for the values and the Manova test did not show differences for the economic preferences (but for the values). Taken together, this justifies using the pooled samples. Furthermore, we also used nationality as a control variable in the regression (Table 2). |
| 11. The authors should keep the reported values to the same decimal places. Sometimes they report up to 2, other times to 3. | We have now changed all decimal places to 2 throughout the paper. |
| 12. The authors should make sure the values they reported in the table are the same as what they reported in the text. Just to give one example, on page 15, 2nd line, they reported an r=-0.283 but the corresponding number in Table 3 is -0.284. There are too many inconsistencies. The burden should fall on the authors, not on the readers. | Thanks for pointing out this mistake. We have thoroughly re-read the manuscript and made sure that the numbers correspond in tables, figures and the text. |
| 13. When the authors said there is a weak correlation, do they base the claim on a small r or a small p-value? The authors should explain their criterion and stick to it consistently throughout. | Done. We now interpret the correlations as small or medium based on Cohen 1992. |
| 14. The authors did not report the results for H7 and H8. But in fact, positive reciprocity does correlate with self-transcendence. Why do they ignore this result? Positive reciprocity also negatively correlates with self-enhancement, by the way. | Corrected, these results are now reported. |
| 15. The author should justify why they do not do multiple comparison corrections for the results they report in Table 3. If the corrections are made, some significant results may become insignificant. | Done. Using the most conservative approach of Bonferroni, we can still retain 7 out of 8 supported hypotheses. We reported this finding in the paper. |
| 16. Shaded color should be explained in the legends of Table 3. | The shades were colors for the positive (green) and negative correlations (red). As many people print papers in black and white, we decided to remove the colors. |
| 17. GPS does include preferences on patience. The authors do not mention patience throughout the manuscript but somehow there is a column on patience in Table 4. | Concerning the variable patience, it is one of the standard variables in the preference survey, and therefore we measured it. However, we did not have any hypothesis for a relation with values and consequently did not preregister any. Therefore, we had not intended to report the results of this variable in the paper, but it had found its way into the draft. In the new version, we have removed it from all tables. In case the reviewers find it relevant, we could consider reporting it after all. |
| 18. On page 20, the last paragraph the authors seem to expect that results in step 2 would be similar to those in step 1. I do not understand their logic. They only calculate correlation coefficients in step 1. But in step 2, they run multiple variable regressions. Logically there is no guarantee that one implies the other. So why should one expect to see similar results in both steps? Because the results are not robust, the authors should address the conceptual difference among the three steps to help the readers understand which step best addresses their research curiosity. | In fact, we do expect that our hypothesized relations between values and economic preferences would come through in the regression as well. The regression is a stricter test than simple bivariate correlations, because it allows for controlling for several variables and associations simultaneously. We used a regression, controlling for sociodemographics and the other values hypothesized to address potential omitted variable bias when testing for a correspondence between values and economic preferences. We now explain this in the results section and in the conclusion to make our test procedure rationale clear. |
| 19. Table A5 is confusing. I don’t understand what “Same Sample all” means though I did notice that the sample size is fixed at 296. | The main table in the paper includes also individuals who did not respond to all questions and we ran an analysis with only those individuals who answered all questions for robustness. We have taken the table out of the paper to avoid confusion, but the data and code to run it are available under the link mentioned above. |
| 20. The title of Table A6 should be changed. The authors are regressing values on preferences here. | Corrected. |
| 21. The shorthands for lower-level values in Table A8 should be explained. | Done, explained. |
| 22. On page 39, several p-values and table number are missing (see XXX and x). | The missing numbers have now been added. |
| 23. Currently all figures are called Fig. 1. See pages 43, 44. | Done. Now there is only two figures in the Appendix. |
| 24. Again, the shorthands for lower-level values on page 44 should be explained. | Explanations have been added where these are mentioned. |
| 25. Overall, the manuscript is not written with enough care. The authors should read the manuscript before they submit their revised version. | Thank you for pointing out in detail where the manuscript is not consistent. We have now rewritten many parts of the manuscript, corrected what needed correcting in line with the comments of the editor and both reviewers, cut unnecessary parts and tables or figures, and tried to improve the writing ourselves and with the help of a native speaker. We hope the manuscript is now much improved in its readability.  Thank you very much for all the useful, constructive and helpful comments! |

Reviewer 2

| Comment | How we addressed it |
| --- | --- |
| 1. The topic is interesting, but its execution has several weaknesses. These concern both the theoretical framework and the methodology. | We tried to address all the comments, see below. |
| 2. Several issues, I think, are not yet well developed in the current manuscript. In addition, the layout is at times a bit sloppy. | We tried to further develop those issues mentioned by the two reviewers and the editor and improved the layout. |
| 3. The focus in the article is on the study of human values and that part of the manuscript is well developed. However, in the literature on behavioral economics mainly older references are used and I miss more recent additions to this literature such as the work by e.g., Thaler and Camerer. | We have now added many more references to recent advances in behavioral economics to the paper, specifically to the motivation section and the section on describing the economic preferences. |
| 4. Page numbers are missing in the manuscript. | We have now added page numbers to the manuscript. |
| 5. On p. 2 it is stated that the values approach works top-down and the behavioral economics approach bottom-up. This may be (is) true, but why is this a reason to bring these streams together? Who will benefit? The economics literature or the values literature? Please elaborate. | Researchers sometimes work in peaceful co-existence instead of building on previous theories (Karl Dieter Opp, personal communication). They may use similar concepts which have different names. Thus, it is fundamental to determine whether economic preferences and basic human values, two very widely used theories, include similar concepts with different names and measures. If not, they may prove themselves to be complementary for explaining behavior. We have now added some discussion in the paper on why it is important to examine relations between concepts in the two theories.  The following has been added to the abstract:  “Could it be that they represent similar motivations, that is, that they are equivalent to each other and mutually exclusive, or are they rather complementary? Finding that they are, rather, complementary may suggest that combining them to explain behavior might be beneficial.” |
| 6. On p. 3 it is stated that the fields use “fundamentally different methods”. Is that true? In more applied fields such as marketing a behavioral economics approach is taken employing experiments and surveys which are also part of the values’ researcher’s toolbox. Please clarify what the differences are. Later in this page it is stated that the respective literatures will be enriched, but how? What may it bring to economists or values researchers? | Thanks for letting us clarify the issue. We have softened the statement a bit, as the reviewer correctly points out that the two literatures in part use similar methodologies, e.g. in marketing, direct questions on preferences may be used). We clarified the main differences between a top-down approach in the values literature approach of trying to get to the broadest most abstract motivation of humans by directly asking them, and the revealed preferences approach that infers preferences through structural equations derived from the choices of individuals.  We examine whether the basic human values and the economic preferences are mutually exclusive or rather complementary. If results suggest that the two approaches are not mutually exclusive but rather complementary, then it may prove useful to combine them for explaining human behavior. This would potentially benefit both disciplines. |
| 7. On p. 7 the hypotheses on e.g., risk, trust, and altruism are not novel for researchers in business economics (see e.g., Keh & Sun, 2008 in JIM; Kumar et al., 2021 in JRCS; Ahmad et al., 2023 IMR). | It is true that there have been studies in the past that connected/identified a link between economic preferences with/and some measure of values more broadly. We have now acknowledged this in the paper. We consider our hypotheses innovative in the sense that we have not identified other studies investigating the relations between Schwartz’s established theory of basic human values (which is one of the most used theories in social psychology to explain behavior) and economic preferences. |
| 8. On p. 10 in the method section the samples are different in age which may result in the samples that differ in the importance of and the variance in their values. Are these students from the same faculty? It is known that topic of study affects values (Bardi et al., 2014). Please provide more information on the students’ background. | Indeed, the samples are different in their composition, with older participants in the Polish sample. Recruitment was done by way of snowballing. In a MANOVA test (footnote 9), we report a test of similarity, where we identified differences in respondents’ values. We also undertook a measurement invariance test for values and found full metric invariance and partial scalar invariance (see Appendix).  However, our goal was to examine relations between values and economic preferences rather than to describe populations. We used two samples to allow a better generalizability of the results and strengthen external validity. Findings for the separate samples are reported in the Appendix. Since the conclusions based on them were essentially similar, and to save space, we reported the results based on the pooled sample in the body of the text. Furthermore, in the regressions we controlled for sociodemographic characteristics (like age, gender, income and nationality) to account for potential compositional effects. |
| 9. P. 10, survey design. The samples are from different countries. Please provide information on assessment of common method variance and invariance testing when appropriate. I think this holds at least for part of the Falk et al. survey. | The economic preference variables based on the Falk et al. survey method are constructed as per the instructions in their paper (in other words, as formative rather than reflective items). We applied their suggested weights to form the economic preference indices to be consistent with how the authors created them. Doing this (rather than estimating factor loadings as would be the case for reflective indicators) would not allow for examining common factors or measurement invariance (Podsakoff et al. 2003). However, we tested for measurement invariance for the values across countries. Partial metric invariance was supported by the data, suggesting that associations are comparable across samples. We now comment on it in footnote 9 and add the table with the full results to the appendix. |
| 10. p. 11. Why is the focus on the four higher-order values? | Shalom Schwartz suggested that we may use broader or more specific values, depending on our research purpose (Schwartz et al. 2012). This study focused on the higher-order dimension because it is the first step in examining associations between economic preferences and basic human values. Future studies may go into more detail examining more specific values. |
| 11. It worries me that in Table 4 the effect of “Other” is large and significant. What does it stand for? | As explained in the Note, “other” stands for the gender selection that is not male or female. We wanted to be inclusive in the survey, but eventually only one person checked this option. When removing this one person from the regression, the results are unaffected, see below. For reasons of completeness, we still use the full data for the main result, but we do no longer display the controls in the main paper, and only in the Appendix.   |
| 12. On p. 17 the focus becomes on MDS. It is not clear what type of MDS is used. Please elaborate on this. The authors mention that they use MDS to visualize the results. Visualization is a nice feature of MDS, but why is the visualization done? What insight does it provide that helps us understand the importance of linking the behavioral economics approach to the values approach? | After reconsideration of the use of MDS models in the papers, we agree that they do not add significantly to the findings and insights beyond the correlations and regressions performed. Therefore, and for the sake of simplicity and shortening the paper, we decided to omit them. |
| 13. In the summary and discussion, I again get confused on who is the target audience of this manuscript. Is it behavioral economists or values researchers, or both? | We have now tried to rewrite the final section, by first elaborating on why finding similarities and differences between the concepts may be to the benefit of both disciplines, and by elaborating in more detail about the limitations of the study. Furthermore, a discussion on the implications of the findings for behavioral economists has been added. |
| 14. I miss a clear statement on what can be learned from the current research. | We concluded that the two approaches are not mutually exclusive but rather complementary. Thus, combining them for explaining human behavior may be beneficial. |
| 15. You may bring results from Table A7, A9 and A10 to the main text, I think this appeals more to economists. | There are two different audiences for this paper: economists and psychologists, who have somewhat different reporting strategies. We decided to remain parsimonious as much as possible in the body of the text but to include additional findings that are not strictly part of the hypotheses testing to the Appendix. Indeed, the first reviewer criticized that too many results are reported. However, if required, we are happy to bring more findings from the Appendix into the main text. |
| 16. Finally, please elaborate the discussion including recent work from both values research and behavioral economics. | We have now elaborated the discussion part and hope that it contributes better to an understanding of the main goals and the added value of the findings. |
| 17. p. 4 last sentence. Value do not “determine” attitudes. There is a correlation, however, there is no causality. | Thank you, corrected. |
| 18. p. 12/13 Please provide information on Germany and Poland separately in the descriptive statistics. Also clarify whether the centring was done per country. The “0” on all dependent variables suggests otherwise. | We now provide information on Germany and Poland as well as regression results for the separate samples in the Appendix. The centering was done around the individual mean across the full sample that was included in the regression. Given the Manova test (footnote 9) mentioned that there is no group difference for economic preferences and that there is partial scalar invariance, we believe this is valid. |
| 19. p. 12 Please change “Gender” in “male or “female” for clarity. Please also check the effect on outcomes when the “3” for gender is excluded. I do not expect many differences, but an outlier may affect results. | See response to comment 11 above. Excluding the “other” gender does not affect the findings. As there are three genders, we cannot rename the variable in the main description to female/male. |
| 20. p. 13 Please provide more insight into the data. How is the distribution of e.g., risk? | We now provide the means, standard deviations, min, and max for all variables. |
| 21. p. 13 why is it unexpected that trust is positively related to conservation? The context of the questions may affect this result (e.g., being in a well-known traditional environment may breed trust). | Indeed, this relation may appear sensible post-hoc. Thus we deleted the word “unexpected” in the results section. |
| 22. p. 14 relationship altruism conservation and openness. Why unexpected? In the charitable giving literature this result is common. | We have now omitted the word “unexpected”. |
| 23. p. 18 Figures 2 and 3 are not in the paper. All Figures are titled “Figure 1”. Likely a typing error. | Thanks for pointing out this mistake. In the new version we have only one figure, and it is in the Appendix. |
| 24. p. 20 last paragraph. Please add references to substantiate the points made. | The MDS is now excluded from the paper. |

Reviewer 3

| Comment | How we addressed it |
| --- | --- |
| 1. As behavioral economic preferences, the authors measure risk-taking, altruism, trust, and positive/negative reciprocity. I agree that they are important factors, but other factors also have been identified as key factors in economic decision-making. A prominent example is time preference (i.e., how patient an agent is). It would be better to explain the reason why the authors focus on risk-taking, altruism, trust, and positive/negative reciprocity (perhaps, is it because time preference seems to be unrelated to values?) | Indeed, time preference (patience) is one of the standard variables in the preference survey, and therefore we measured it. However, we did not have any hypothesis for a relation with values. We had not intended to report the results of this variable in the paper, but it had found its way into the draft. We have removed it from all tables in the new version. In case the reviewers find it relevant, we could consider reporting it anyway. |
| 2. Related to the first point, the rightest column in Table 4 contains patience as a dependent variable. Does it mean that the survey module includes questions on time preference? If so, this point should be explained and the analysis of them should be included in the main text. In addition, would there be some other behavioral economic preferences measured but not explicitly mentioned in the paper? If so, they should be also explained. | Please see previous answer. We have not collected any additional economic preference that is unreported. We have, however, collected the big five personality traits, but again without any pre-registered hypotheses – and therefore do not report any analysis on them. We find it prudent to stick with the analyses we had foreseen, but again could include those additional tests in the Appendix if requested. |
| 3. The authors mention "the complete survey and the item construction can be found in the Supporting Information," but I could find them in the supporting information. The availability of the survey questions is important to enhance the replicability of the research. It would be better to present them in the supporting information. (In case it was presented but I missed them, I'm very sorry for it). | All data, codes and the questionnaires are now available under: <https://osf.io/v3kqt/?view_only=69213a56704647ae910a10cc70fcada5> |
| 4. I missed how incomes were coded. According to Table 2, it seems that they were measured on a 6-point scale, but it would be better to explain the details (I found how gender was coded). | Income is measured using the standard question from the European Social Survey:  What best describes how you assess your current household income? With the current income  I/we can...  K1.  1 live comfortably  2 get by  3 have difficulty getting by  4 have great difficulty getting by  5 (Answer refused)  6 (Do not know)  Categories 5 and 6 are coded as missing. |
| 5. A sample in Germany is the student sample, while that in Poland is not the student one. Is the latter one a representative sample? How were they recruited? It would be better to explain these points. | The samples are convenience samples recruited using the snowball technique. None was representative. |
| 6. Related to these points, I have several questions (it would be better to explain them either in the main text or in the supporting info.): a.) I understand that this survey is not a monetary incentivized one, but did you pay some money for completing the survey or was it perfectly volunteered? | Participants volunteered for the survey and were not remunerated in monetary or other form. We have now added this information in the Methods section. |
| 7. b.) At the beginning of the method section, the authors mention "a student sample from the University of Cologne". I expect that this is a German sample, and it might be better to explain this explicitly. | While this is a sample from the University of Cologne in Germany, the nationality of respondents was not exclusively German. Therefore, in the regressions we controlled for Nationality, rather than the country sample when pooling the samples. In total, the sample consisted of 211 Polish nationals, 120 German nationals and 8 persons of other nationalities. We have added this information to the methods section. |
| 8. c.) In the conclusion, the authors mention "There are several limitations to our study, the first of which being that we are making inferences using student samples." From this sentence, one might have an expression that both Poland and German samples were students. It might be better to note that the Polish samples were not students. | Thank you for pointing this out. Indeed, our concern was not correctly formulated. We now refer to convenience samples, although our limitation remains an issue as convenience samples are not representative. |
| 9. d.) If this survey were a monetary-incentivized laboratory experiment, I would understand that only student samples are convenient. However, this is just a survey and convenient samples that are more diverse socio-demographically (e.g., amazon M-Turk samples in the US). It might be useful to explain why the authors chose student samples. | We mainly relied on this simple recruitment method for convenience and as the reviewer correctly pointed out, there were not only students among the respondents. We were not interested in descriptive evidence but in associations, for which we did not necessarily need representative or particularly diverse samples. However, we believe that having samples with different characteristics from two different countries provides us with heterogeneity and offers higher external validity for the findings. |
| 10. In addition to presenting correlation coefficients, it might be also useful to show scatter plots where the x-axis is BPP and the y-axis is value, because it visualizes that the correlation is only moderate. | We thank the reviewers for suggesting this plot. As the other reviewers have already mentioned that they find the amount of figures and tables overwhelming, we placed this one in the Appendix as Fig A9.    Additionally, if we look at the within and across measure variations, we find that correlations within the economic measures is on average 0.09 (for the absolute value of the correlations), and 0.37 for values (0.17 for the 19 lower-level values, untabulated) and 0.13 for the cross-measure correlations (0.10 for lower-level values). The maximum correlation for economic measures is 0.28, for values 0.74 (0.53 for lower-level values) and for cross correlations 0.32 (0.28 for lower-level values). Thus, while any measures within a scientific tradition may have a certain correlation (Albanese et al. 2017), there is no reason why this should be the case across disciplinary boundaries. The value correlations are particularly high as they cover a continuum (and based on the theory, all values are desirable), but the fact that cross-measure correlations are higher than the ones within the economic measures shows that this relation is more than incidental. |
