# Supplementary material for: One and the same? How similar are basic human values and economic preferences
Source: PLoS One. 2024 Feb 15;19(2):e0296852. doi: 10.1371/journal.pone.0296852 (PMC10868778; doi:10.1371/journal.pone.0296852)
Supplement: Attachment — Submitted filename: Response to reviewers.docx [file pone.0296852.s014.docx]

Reviewer comments and how we addressed them

Reviewer 3

| Comment | How we addressed it |
| --- | --- |
| I appreciate the authors' effort in revising the manuscript. I think the points raised by my referee report were addressed adequately. | We would like to thank the reviewer for the thorough review of the revised manuscript. The review helped us considerably to further improve the manuscript. |
| 1. Abstract "they are equivalent to each other and mutually exclusive, or are they rather complementary": In mathematics, A and B are mutually exclusive if  A ∩ B is empty. Therefore, saying that economic preferences and values are mutually exclusive seems to indicate that they are totally different concepts. However, in the above sentence, it is used to indicate that they are similar concepts. This is quite confusing for me. | Thank you for spotting this inconsistency. We have indeed overlooked it. Now the abstract reads: “But how similar are they? Finding that they differ may suggest that…” (we deleted the sentence mentioned by the reviewer, because it is confusing and inconsistent). We corrected this also on page 4. |
| 2. Page 5, second paragraph: "We try to)" -> "We try to" | Corrected. |
| 3. Page 8, footnote 2: "preference patience" -> "preference for patience"  "we did not" ->". Thus, we did not" | Corrected. |
| 4. Page 13 "We collected data using convenience samples in two countries, Poland and Germany: 212 from Poland (123 females, 89 males, mean age 34.0 years, all of Polish nationality) and a student sample of..." It would be better to explicitly mention that the Poland sample is not a student sample. | Done: “…Poland and Germany: a non-student sample of 212 from Poland (123 females…”. |
| 5. Page 13: In the reply letter, the authors said "We were not interested in descriptive evidence but in associations, for which we did not necessarily need representative or particularly diverse samples." I agree that this could serve as a justification for using convenience samples in the study. However, this point is not explained in the method section (page 13). It would be better to explain this point. Relatedly, there are several studies reporting that associations between behaviors/attitudes are similar even when we compare convenience samples (including student ones) and representative samples (e.g., Snowberg, E., & Yariv, L. (2021). Testing the waters: Behavior across participant pools. American Economic Review, 111(2), 687-719.) Citing such existing studies may be worthwhile. | Done: P. 13: “Convenience samples were sufficient for our purpose, because we were not interested in descriptive evidence but in associations, for which we did not necessarily need representative or particularly diverse samples. Several studies have shown that associations between behaviors or attitudes are similar even when comparing them across convenience samples (including student ones) and representative samples (see, e.g., Snowberg, & Yariv [71]).” |
